# Smooth muscle–derived adventitial progenitor cells direct atherosclerotic plaque composition complexity in a Klf4-dependent manner

**DOI:** 10.1172/jci.insight.174639

**Published:** 2023-11-22

**Authors:** Allison M. Dubner, Sizhao Lu, Austin J. Jolly, Keith A. Strand, Marie F. Mutryn, Tyler Hinthorn, Tysen Noble, Raphael A. Nemenoff, Karen S. Moulton, Mark W. Majesky, Mary C.M. Weiser-Evans

**Affiliations:** 1 Department of Medicine, Division of Renal Diseases and Hypertension,; 2 Integrated Physiology PhD Program,; 3 School of Medicine, Consortium for Fibrosis Research and Translation,; 4 Medical Scientist Training Program, University of Colorado School of Medicine, Anschutz Medical Campus, Aurora, Colorado, USA.; 5 Biomedical Sciences and Biotechnology MS program, University of Colorado Graduate School, Anschutz Medical Campus, Aurora, Colorado, USA.; 6 Department of Medicine, Division of Cardiology, University of Colorado School of Medicine, Anschutz Medical Campus, Aurora, Colorado, USA.; 7 Center for Developmental Biology & Regenerative Medicine, Seattle Children’s Research Institute, Seattle, Washington, USA.; 8 Departments of Pediatrics, Laboratory Medicine & and Pathology, University of Washington, Seattle, Washington, USA.; 9 Cardiovascular Pulmonary Research Program, University of Colorado Anschutz Medical Campus, Aurora, Colorado, USA.

**Keywords:** Stem cells, Vascular Biology, Adult stem cells, Atherosclerosis

## Abstract

We previously established that vascular smooth muscle–derived adventitial progenitor cells (AdvSca1-SM) preferentially differentiate into myofibroblasts and contribute to fibrosis in response to acute vascular injury. However, the role of these progenitor cells in chronic atherosclerosis has not been defined. Using an AdvSca1-SM cell lineage tracing model, scRNA-Seq, flow cytometry, and histological approaches, we confirmed that AdvSca1-SM–derived cells localized throughout the vessel wall and atherosclerotic plaques, where they primarily differentiated into fibroblasts, smooth muscle cells (SMC), or remained in a stem-like state. Krüppel-like factor 4 (*Klf4*) knockout specifically in AdvSca1-SM cells induced transition to a more collagen-enriched fibroblast phenotype compared with WT mice. Additionally, *Klf4* deletion drastically modified the phenotypes of non–AdvSca1-SM–derived cells, resulting in more contractile SMC and atheroprotective macrophages. Functionally, overall plaque burden was not altered with *Klf4* deletion, but multiple indices of plaque composition complexity, including necrotic core area, macrophage accumulation, and fibrous cap thickness, were reduced. Collectively, these data support that modulation of AdvSca1-SM cells through KLF4 depletion confers increased protection from the development of potentially unstable atherosclerotic plaques.

## Graphical abstract

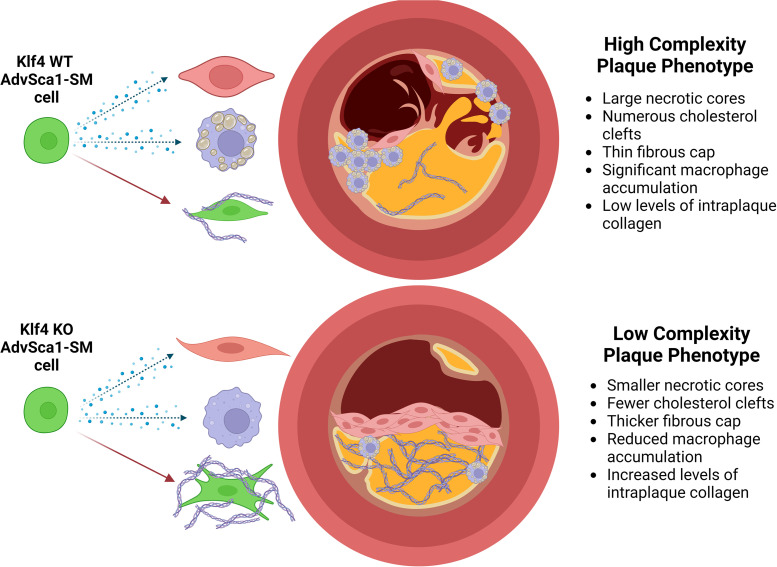

## Introduction

Atherosclerosis is a complex inflammatory condition and the major driver of cardiovascular disease, a spectrum of diseases resulting in approximately 32% of global deaths (1, 2). Approaches to management of atherosclerosis include lipid-lowering and antiinflammatory medications. Unfortunately, statins fail to fully resolve CVD risk, and despite intense lipid lowering, many patients have residual risks. Additionally, antiinflammatory treatments have limitations due to cost and the need for chronic use, which carries increased risk of infection and sepsis. Thus, additional therapeutic approaches are needed to directly target the vascular wall cells in atherosclerotic lesions (3–8). Surprisingly, few if any therapies focus on the pathological mechanisms of resident vascular cells, and this may provide insights for future therapy beyond current care.

Historically, research in the field of atherosclerosis has centered on the role of the innermost layer of the blood vessel, the intima, in driving atherosclerosis progression through endothelial dysfunction, lipid accumulation/oxidation, and macrophage infiltration (1, 3–5). More recently, lineage-tracing studies in murine models and human atherosclerotic tissues have defined the important role vascular smooth muscle cells (SMC) play in disease progression. However, while less frequently studied, it has been well recognized that cells in the outermost layer of the vessel, the adventitia, are critical contributors to early stages in the pathogenesis of atherosclerosis. Furthermore, the “outside-in” hypothesis posits that expansion of adventitial microvessels, the vasa vasorum (VV), acts as an early and potent driver of plaque progression by facilitating inflammatory cell infiltration and tertiary lymphoid organogenesis and by supplying the oxygen/nutrient needs of the growing plaque (6–10).

Recent research has demonstrated that the adventitia is highly dynamic and home to a wide variety of cells, including fibroblasts, leukocytes, and resident vascular progenitor cells (7, 11–13). Our group discovered a subpopulation of these adventitial progenitor cells (AdvSca1-SM cells; adventitial location, Sca1 expression, SMC origin) derived from mature, contractile SMC that undergo KLF4-dependent reprogramming, thus transitioning into a multipotent progenitor (14). Subsequent research demonstrated that maintenance of the AdvSca1-SM stemlike phenotype is dependent on continuous KLF4 activity and that AdvSca1-SM cells preferentially differentiate into myofibroblasts to contribute to perivascular fibrosis in response to acute vascular injury (15, 16). However, the contribution of AdvSca1-SM cells to chronic diseases such as atherosclerosis has not been established.

In this study, we characterized the multifaceted contributions of AdvSca1-SM cells to atherosclerosis. Immunofluorescence microscopy demonstrated that AdvSca1-SM–derived cells are found throughout the adventitia, media, and atherosclerotic plaque. Using single-cell RNA sequencing (scRNA-Seq), we established the major differentiation trajectories of AdvSca1-SM–derived cells as well as their ability to communicate with other cells in the plaque microenvironment. Surprisingly, we found that AdvSca1-SM cell–specific KO of *Klf4* altered both the differentiation trajectories of AdvSca1-SM cells and the transcriptomic profiles of non-AdvSca1-SM cell–derived SMC and macrophages, leading to increased fibrous cap thickness and intraplaque collagen deposition with corresponding reductions in necrotic core area, cholesterol crystal accumulation, and macrophage recruitment. These findings indicate that AdvSca1-SM cells are major regulators of atherosclerotic plaque progression and that KLF4-dependent phenotypic transitions of both AdvSca1-SM cells and other vascular cells are critical to plaque complexity, thus highlighting a potential future therapeutic target.

## Results

### Induction of atherosclerosis in AdvSca1-SM cell lineage mice.

**Figure 1:**
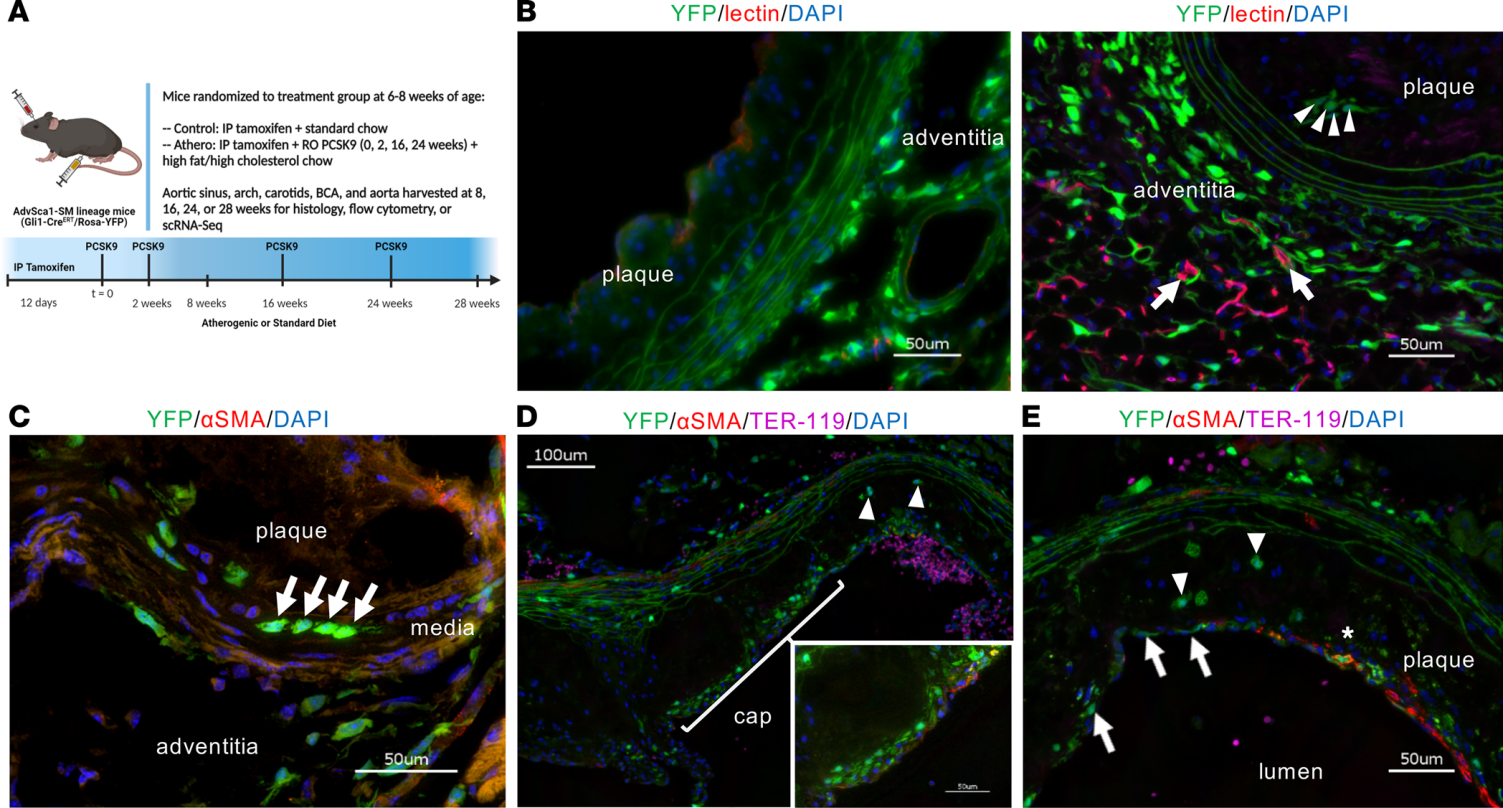
AdvSca1-SM cell–derived YFP^+^ cells are distributed throughout the vascular wall and atherosclerotic plaque. (**A**) Schematic of experimental approach. (**B**) A subset of animals were injected i.v. with fluorescently labeled *Griffonia simplicifolia* lectin I (GSL I) isolectin B4 5 minutes prior to sacrifice to label functional vasculature in vivo. Representative immunofluorescence of aortic root sections from 24-week plaques (*n* = 6) stained for YFP (AdvSca1-SM and AdvSca1-SM–derived cells; green); lectin (red); DAPI for all cell nuclei (blue). Arrows indicate functional adventitial microvasculature (lectin labeled) surrounded by YFP^+^ cells; arrowheads indicate intraplaque YFP^+^ cells. (**C**) Representative immunofluorescence image of 28-week aortic root plaque (*n* = 5) stained for αSMA (red) and YFP (green). Arrows indicate YFP^+^ medial cells. (**D** and **E**) Aortic root slides from 24-week plaques (*n* = 10) stained for YFP (green), αSMA (red), Ter-119 (magenta), and DAPI (blue). Low-power and high-power insert (**D**) show YFP^+^ cells in the cap of the plaque. (**E**) YFP^+^ cells in the plaque cap (arrows), YFP^+^/αSMA^+^ cells in the plaque cap (asterisk), and YFP^+^ cells in the body of the plaque (arrowheads). Scale bars: 50 μm, 100 μm (low-power **D**).

Our previous research indicated a preferential differentiation of AdvSca1-SM cells into myofibroblasts in the setting of unilateral carotid ligation, a well-established model for acute vascular injury, neointima formation, and adventitial remodeling (15, 16). In these experiments, AdvSca1-SM cells were integral to adventitial remodeling and arterial fibrosis. However, the role of AdvSca1-SM cells in chronic vascular diseases, specifically atherosclerosis, has not been established. To elucidate the role of these adventitial progenitor cells in atherosclerosis, we utilized the AdvSca1-SM cell lineage tracing mouse model we previously developed and validated (Gli1-Cre^ERT^/Rosa26-YFP) (15). Compared with other cells in the vasculature, AdvSca1-SM cells express high levels of *Gli1*. Upon tamoxifen injections, the tamoxifen-inducible Gli1-Cre recombinase is activated, and AdvSca1-SM cells and AdvSca1-SM–derived cells are selectively and permanently labeled with the YFP reporter; a complete lack of medial SMC or intimal cell labeling confirms the specificity of this system. After cell labeling, AdvSca1-SM lineage mice were injected with a gain-of-function mutant PCSK9-AAV to knock down LDL receptors and were placed on a high-fat/high-cholesterol diet for 8–28 weeks to induce hypercholesterolemia and atherosclerotic plaque formation, as previously described (Figure 1A and Supplemental Figure 2; supplemental material available online with this article; https://doi.org/10.1172/jci.insight.174639DS1) (17, 18).

### AdvSca1-SM–derived cells are found throughout the vessel wall and plaque in both early- and late-stage atherosclerosis.

We first sought to determine the spatial distribution of AdvSca1-SM cells and their progeny within the vessel wall and plaque. The majority of published studies of atherosclerosis progression primarily focused on lipid accumulation and monocyte recruitment on the intimal surface of the vessel (1, 3, 5). However, additional work has demonstrated the integral role of the VV in driving plaque progression (1, 3–9). Moreover, using an in vivo Matrigel plug assay, we previously demonstrated the ability of AdvSca1-SM cells to form microvasculature, raising the question of whether AdvSca1-SM cells could be involved in the expansion of the adventitial microvasculature in the setting of atherosclerosis (14). To investigate this possibility, a subset of mice after 16 or 24 weeks of atherogenic diet were i.v. injected with fluorescently conjugated isolectin B4 to label functional vasculature. Using this approach, we detected microvessels in the adventitial area of the aortic root. Similar to our findings in acute vascular injury, there was an expansion of YFP^+^ AdvSca1-SM cell–derived cells in the adventitia, with many frequently associated with the adventitial microvasculature, suggesting a neovascularization role for AdvSca1-SM cells in atherosclerosis and supporting our previous Matrigel plug experiments (Figure 1B). While the majority of YFP^+^ cells were found in the adventitia, they were also observed in the medial layer of the aortic root (Figure 1C). Finally, we observed YFP^+^ cells localized to the plaque itself, both via formation of the fibrous cap (Figure 1, D and E) as well as within the core of the plaque. These data indicate roles for AdvSca1-SM cells throughout the vascular wall in the context of chronic atherosclerosis and suggest multifaceted contributions to atherogenesis.

### scRNA-Seq defines shifts in vascular cell phenotypes in atherosclerosis.

**Figure 2:**
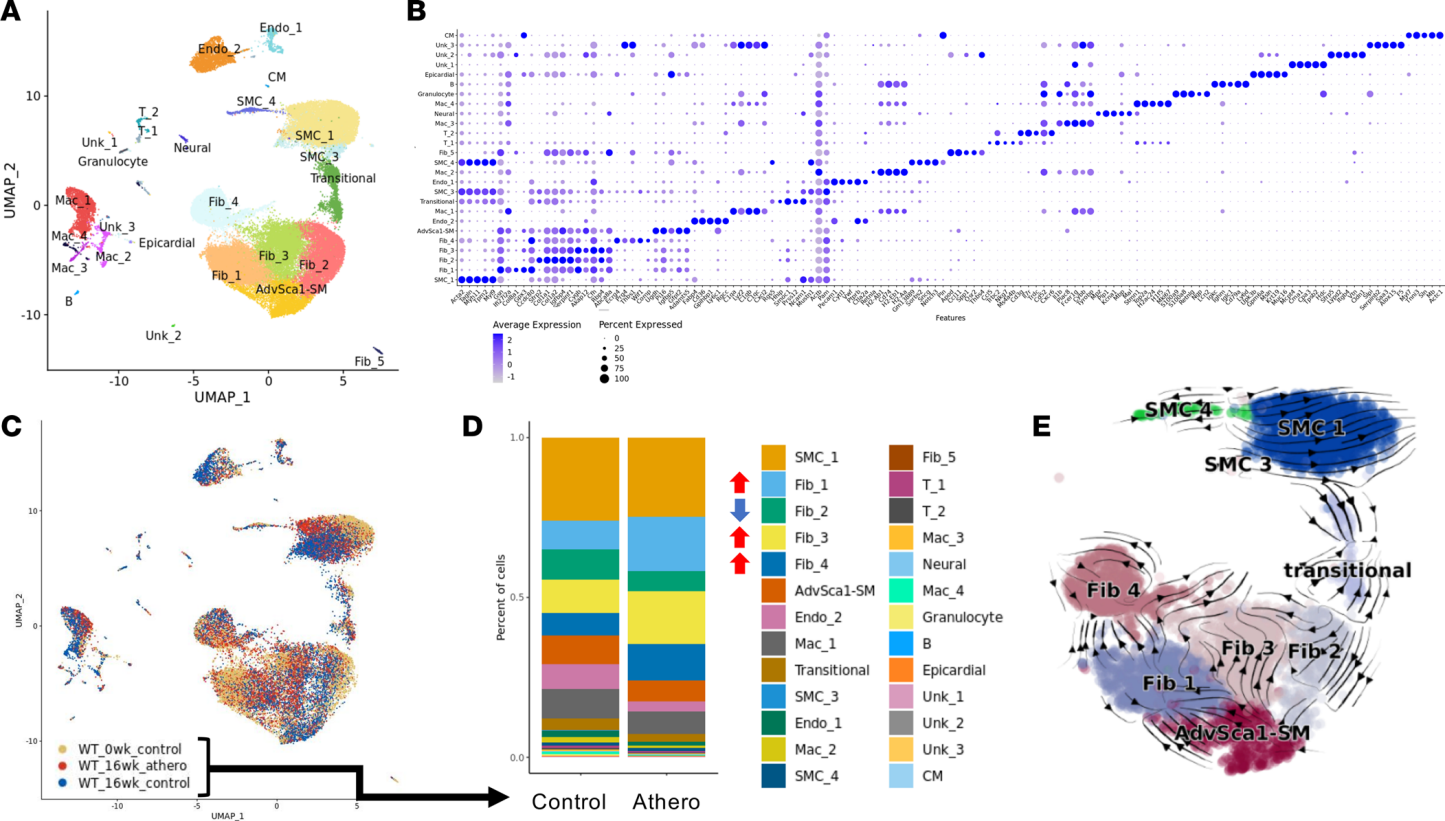
scRNA-Seq analysis demonstrates major shifts in vascular cell types in the setting of atherosclerosis. The aortic sinus, aortic arch, brachiocephalic artery, and carotid arteries were harvested at baseline and after 16 weeks of normal or high fat chow and processed for scRNA-Seq; 3 mice per condition were pooled for analysis. (**A**) UMAP of all cells that passed quality control. Cluster identity was assigned using representative gene expression profiles. (**B**) Dot plot showing the top 5 unique genes that define each cluster. (**C**) UMAP of all cells from baseline, 16-week control, and 16-week atherogenic samples. (**D**) Stacked bar plot of YFP^+^ and YFP^–^ cells from 16-week control and atherogenic samples. Arrows indicate increases (red) or decreases (blue) in the cell population as a result of atherosclerosis. (**E**) RNA velocity analysis of all cells after 16 weeks of atherogenic diet.

Given the wide distribution of AdvSca1-SM cell–derived YFP^+^ cells in the vascular adventitia, media, and atherosclerotic plaque, we used unbiased scRNA-Seq to define the possible functions of AdvSca1-SM cells in atherosclerosis. Arteries from AdvSca1-SM lineage tracing mice at both baseline (after tamoxifen treatment) and after 16 weeks of either control or atherogenic conditions were harvested, processed into single-cell suspensions, and sorted by FACS based on YFP expression; YFP^+^ and YFP^–^ cells were subjected to scRNA-Seq. It is important to note that entire aortae, BCA, carotid arteries, and aortic root with both plaque and nonplaque regions were combined to obtain sufficient cells for sequencing. Data sets from all samples were combined, passed through quality control measures, and processed using the Seurat pipeline, generating a total of 26 cell clusters (Figure 2A and Supplemental Figure 4). Gene expression profiles were used to assign identities to the different clusters, and the top 5 differentially expressed genes per cluster are shown in Figure 2B. As expected, we detected shifts in cell populations between baseline and 16 weeks of control or atherogenic treatment (Figure 2C). We then examined the changes in cell populations between mice on control or atherogenic diet after 16 weeks of treatment. The clusters that showed the most variability as a result of atherosclerosis were the fibroblast clusters (Fib_1, Fib_2, Fib_3, Fib_4). Specifically, we observed increases in the size of Fib_1, Fib_3, and Fib_4 clusters, along with a decrease in Fib_2 (Figure 2D). We also observed a slight decrease in the AdvSca1-SM cell cluster, suggesting an increased pattern of differentiation into other cell types in the setting of atherosclerosis. Since AdvSca1-SM cells are multipotent progenitor cells and don’t represent a static cell population, RNA velocity analysis was used to predict future cell transition patterns, as described previously (19). Validating our previous findings, the streamplots indicate marked differentiation away from the most stemlike state, the AdvSca1-SM cell cluster, and into other cell types (Figure 2E). These data are in agreement with previous scRNA-Seq data showing shifts in cell phenotype with atherosclerosis progression and confirm a transition of AdvSca1-SM cells away from a stemlike phenotype (20–24).

### AdvSca1-SM cells preferentially differentiate into fibroblasts or remain in a stem-like state in atherosclerosis, with minor contribution to SMC populations.

**Figure 3:**
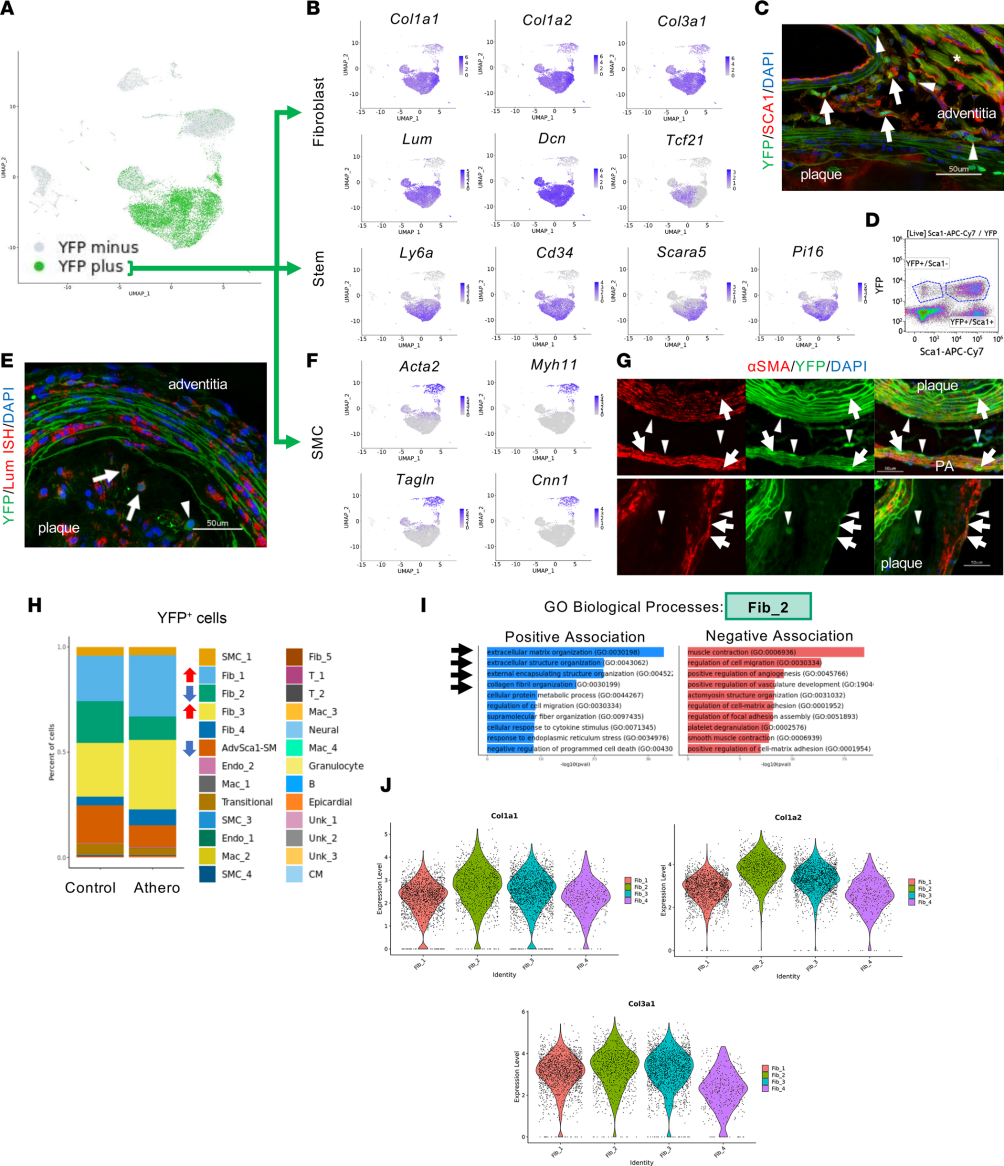
AdvSca1-SM cells differentiate into fibroblasts or SMCs or remain in a stem-like state in atherosclerosis. (**A**) Feature plots showing distribution of YFP^+^ and YFP^–^ cells on the UMAP. (**B**) Feature plots of major fibroblast (*Col1a1*, *Col1a2*, *Col3a1*, *Dcn*, *Lum*, and *Tcf21*) and stem cell (*Ly6a*/Sca1, *Cd34*, *Scara5*, *Pi16*) markers in YFP^+^ cells. (**C**) Representative aortic root image from 24-week plaques (*n* = 10) stained for YFP (green), Sca1 (red), and DAPI (blue). Arrows indicate YFP^+^ Sca1^+^ cells in the adventitia; arrowheads indicate YFP^+^ Sca1^–^ cells. * = cardiomyocyte autofluorescence. (**D**) Aortic sinus, aortic arch, brachiocephalic artery, and carotid arteries from 16-week control (*n* = 5) or atherosclerotic (*n* = 11) mice were processed for flow. Representative image of a YFP and SCA1 density plot from one atherogenic animal. (**E**) Double RNAscope and immunofluorescence image of an aortic root from 24-week atherogenic animals showing lumican (*Lum*; fibroblast cell marker; red) mRNA and YFP (green). Arrows indicate YFP^+^/lumican^+^ cells; arrowheads indicate YFP^+^/lumican^–^ cells; *n* = 3. (**F**) Feature plots of SMC genes (*Acta2*, *Cnn1*, *Mhy11*, *Tagln*) in YFP^+^ cells. (**G**) 24-week aortic root plaques (*n* = 10) stained for YFP (green), αSMA (red), and DAPI (blue). Arrows indicate YFP^+^ αSMA^+^ cells forming the fibrous cap of the plaque or contributing to the media. Arrowheads indicate YFP^+^ αSMA^–^ cells. PA = pulmonary artery; *n* = 11. (**H**) Stacked bar plot showing phenotypic shifts in YFP^+^ cells between 16-week control and atherogenic samples. Arrows indicate increases (red) or decreases (blue) in the cell population with atherosclerosis. (**I**) GO Biological Process between the Fib_2 cluster and all other cell clusters. Arrows indicate the top 4 processes positively associated with Fib_2. (**J**) Violin plots demonstrating the stronger collagen gene signature (*Col1a1*, *Col1a2*, and *Col3a1*) in Fib_2 compared with Fib_3. All scale bars are 50 μm.

Leveraging our AdvSca1-SM lineage tracing mouse model, we investigated specific changes in the YFP^+^ AdvSca1-SM and AdvSca1-SM–derived cell populations in the setting of atherosclerosis. YFP^+^ cells were primarily found in the stemlike AdvSca1-SM, and fibroblast-like Fib_1, Fib_2, and Fib_3 clusters (Figure 3A). These clusters expressed high levels of myofibroblast (*Tcf21*) and extracellular matrix (ECM) markers (*Col1a1*, *Col1a2*, *Col3a1*, *Lum*, *Dcn*), and stem cell markers (*Ly6a*, *Cd34*, *Scara5*, *Pi16*) (Figure 3B). However, the distribution of these markers varied between the clusters, with the AdvSca1-SM cell cluster representing the most stem-like phenotype, Fib_2 having elevated collagen gene expression compared with the other fibroblast clusters, and Fib_1 having the highest expression of myofibroblast marker *Tcf21*.

As noted in the scRNA-Seq data, a substantial population of YFP^+^ cells do not differentiate into other cell types but rather retain expression of a stemlike phenotype, as shown by expression of the stem cell marker *Ly6a* (SCA1). Confirming these data, aortic roots stained for YFP and SCA1 exhibited a large population of YFP^+^ SCA1^+^ cells in the adventitia (Figure 3C). Flow cytometry analysis of animals after 16 weeks of atherogenic diet also supported these findings, demonstrating a considerable proportion of YFP^+^ SCA1^+^ stemlike AdvSca1-SM cells (Figure 3D). As noted above, both scRNA-Seq and flow experiments were performed on whole arterial tissue, as compared with immunofluorescence analysis, which focused specifically on plaque-affected regions of the vasculature. Since atherosclerosis is a focal disease, many of the stemlike AdvSca1-SM cells detected in scRNA-Seq and flow cytometry are likely from regions of uninvolved tissue.

We also confirmed the differentiation of YFP^+^ cells into fibroblasts using immunofluorescence and RNAscope microscopy. Investigation of aortic roots revealed YFP^+^ cells in both the adventitia and plaque that coexpress the fibroblast marker, lumican (Lum) (Figure 3E). Please note that not all Lum^+^ cells coexpressed YFP; however, these data further support that AdvSca1-SM have the potential for differentiation toward a fibroblast phenotype in the setting of atherosclerosis. In addition to differentiation toward a fibroblast phenotype or remaining as a stem cell, we detected a subset of YFP^+^ AdvSca1-SM cells differentiating toward a SMC fate. YFP^+^ cells found in the SMC clusters were found to express genes specific to mature vascular SMC, including *Acta2*, *Myh11*, *Cnn1*, and *Tagln* (Figure 3F). This differentiation profile suggested by scRNA-Seq was then confirmed using immunofluorescence microscopy, in which we identified YFP^+^/αSMA^+^ cells in the plaque fibrous cap and aortic media (Figure 3G). Finally, we identified extremely rare differentiation of AdvSca1-SM cells into other cell types, including macrophages, adipocytes, and endothelial cells (Supplemental Figure 6).

As expected, the proportion of YFP^+^ cells in each of the cell clusters was altered in mice on an atherogenic diet compared with controls. Specifically, there were increased percentages of YFP^+^ cells in Fib_1 and Fib_3 clusters with a corresponding decrease in Fib_2 and AdvSca1-SM cell clusters in the setting of atherosclerosis (Figure 3H). To elucidate the functional importance of these cell shifts, we further characterized the phenotypes of the different fibroblast clusters using GO Biological Process pathway analysis. We determined that, compared with all other cell clusters, Fib_2 was enriched for pathways related to ECM and collagen organization (Figure 3I). These findings were confirmed by comparing average expression of collagen genes between the various fibroblast clusters, with Fib_2 showing the highest expression of these genes (Figure 3J). Collectively, these data indicate that chronic hyperlipidemia–induced atherosclerosis promotes activation and a shift in the phenotype of AdvSca1-SM cells predominantly toward a variety of fibroblast-like cells as well as the contribution of AdvSca1-SM cells to adventitial remodeling and plaque formation.

### Dynamic exchange between AdvSca1-SM cells and SMC in the setting of atherosclerosis.

**Figure 4:**
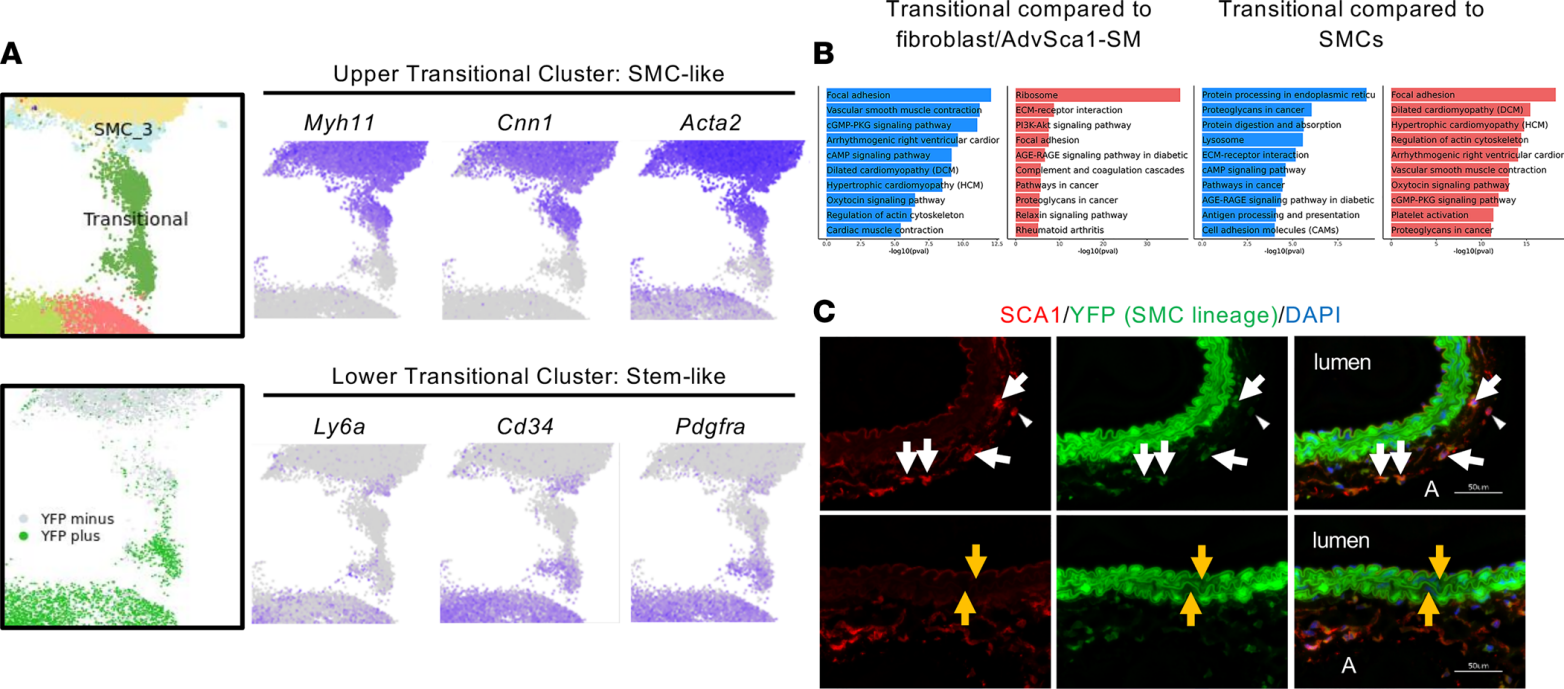
Dynamic, bidirectional differentiation between AdvSca1-SM cells and SMC in the setting of atherosclerosis. (**A**) The transitional cluster consists of YFP^+^ and YFP^–^ cells and exhibits features of both SMC and AdvSCa1-SM cells, roughly divided in half (bottom). Feature plots show that the upper portion of the transitional cluster expresses more contractile SMC genes (*Acta2*, *Myh11*, *Cnn1*), whereas the lower portion expresses more mesenchymal stem cell markers (*Ly6a*, *Cd34*, *Pdgfra*). (**B**) KEGG pathway differences between the transitional cluster and the AdvSca1-SM/fibroblast clusters or SMC clusters. (**C**) Representative image of 14-week atherogenic brachiocephalic artery (*n* = 14) from SMC lineage tracing mice (Myh11-CreERT^+/–^/Rosa26-YFP^+/+^) stained for YFP (green; expression indicates SMC, not AdvSca1-SM). Arrows indicate adventitial YFP^+^ Sca1^+^ cells; arrowheads indicate adventitial progenitors not expressing YFP (YFP^–^/Sca1^+^); orange arrows indicate DAPI^+^ cells in the vessel media that are negative for YFP. A, adventitia. Scale bar = 50 μm.

RNA velocity analysis of our data (Figure 2E) revealed that, not only do AdvSca1-SM cells differentiate away from their most stem-like state and into other cell types, but a variety of cell transitions occur in the fibroblast and SMC clusters. Of particular interest was the transitional cluster, which occupies Uniform Manifold Approximation and Projection (UMAP) space between SMC and AdvSca1-SM/fibroblast clusters and shows a bidirectional differentiation trajectory. The first trajectory demonstrated a shift from AdvSca1-SM/fibroblast clusters toward a SMC phenotype, a trajectory that we confirmed using immunofluorescence imaging for αSMA (Figure 3, F and G). However, the RNA velocity analysis also suggested a reverse trajectory from non–AdvSca1-SM–derived mature SMC (YFP^–^) toward the AdvSca1-SM/fibroblast–like clusters. Further characterization of this transitional cluster revealed 2 unique subpopulations, (a) an upper cluster, which expresses more SMC genes and is largely YFP^–^, and (b) a lower cluster, which expresses more stem/fibroblast genes and is largely YFP^+^ (Figure 4A). Further supporting the identity as an intermediate or transitional cell population, KEGG pathway analysis of the transitional cluster showed it to be less contractile than mature SMC clusters but more contractile than the fibroblast/AdvSca1-SM clusters (Figure 4B). These data support the reprogramming of YFP^–^ mature SMC toward a stemlike AdvSca1-SM phenotype, consistent with our original discovery of AdvSca1-SM cells (14). To confirm this, we exposed SMC lineage tracing mice (Myh11-Cre^ERT^/Rosa26-YFP) to tamoxifen to label SMC, and we then put them on the same atherogenic regimen as the AdvSca1-SM lineage tracing mice. We found that SMC-derived YFP^+^ cells were found in the vascular adventitia, where they coexpressed SCA1, indicating the transition of a mature, contractile SMC into multipotent progenitor cells (Figure 4C). Interestingly, we also observed YFP^–^ cells (i.e., non-SMC–derived) within the media of the vessel (Figure 4C). These data demonstrate the dynamic nature of these adventitial progenitor cells and support a mechanism whereby SMC maintain and replenish the resident AdvSca1-SM stem cell pool while SMC-derived AdvSca1-SM cells redifferentiate toward a SMC fate to contribute to vessel repair/homeostasis.

### Depletion of stemness transcription factor KLF4 specifically in AdvSca1-SM cells alters their differentiation trajectory in atherosclerosis.

**Figure 5:**
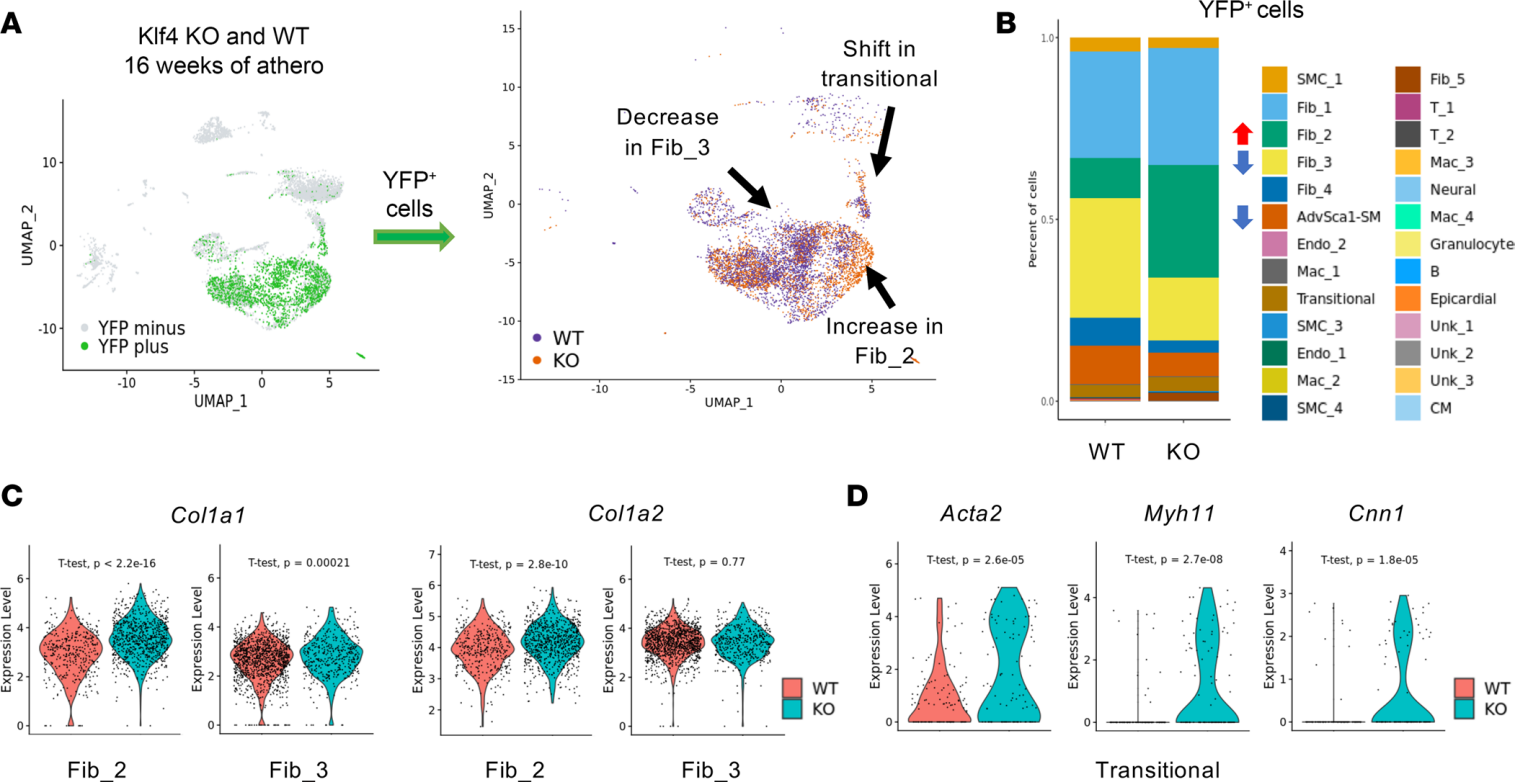
Depletion of KLF4 in AdvSca1-SM cells modifies the fate of YFP^+^ cells in the setting of atherosclerosis. Arteries from 16-week WT and Klf4 KO AdvSca1-SM lineage atherogenic mice were processed for scRNA-Seq. (**A**) Left: UMAP projection of YFP^+^ and YFP^-^ cells from WT and Klf4 KO AdvSca1-SM cell lineage tracing mice. Right: UMAP projection showing the different fates of YFP^+^ cells in atherosclerosis as a consequence of depleting KLF4 in AdvSca1-SM cells. Arrows indicate major phenotypic shifts in KO mice. (**B**) Stacked bar graph of YFP^+^ cells from WT and Klf4 KO mice after 16-week atherogenic diet. Arrows indicate increases (red) or decreases (blue) in cell populations as a function of Klf4 depletion. (**C**) Violin plots showing higher expression of ECM related genes (*Col1a1*, *Col1a2*) in YFP^+^ Fib_2 cluster from Klf4 KO mice compared to WT mice after 16 weeks of atherogenic diet. *Col1a1* is also higher in YFP^+^ Fib_3 cluster cells from Klf4 KO mice compared to WT mice, but there is no difference with *Col1a2* in this cluster. (**D**) Violin plots showing higher expression of SMC contractile genes (*Acta2*, *Myh11*, *Cnn1*) in YFP^+^ Transitional cluster cells from Klf4 KO mice compared to WT mice after 16 weeks of atherogenic diet.

Having established the contribution of AdvSca1-SM cells to atherosclerosis progression, we sought to determine the effect of genetic modulation of these cells on plaque progression and complexity. Given the integral function of KLF4 in the SMC–to–AdvSca1-SM cell transition and maintenance of the progenitor phenotype, KLF4 was selectively depleted in AdvSca1-SM cells as we previously published (14, 15). Following tamoxifen treatment to induce YFP reporter knock-in and *Klf4* KO (in Klf4^fl/fl^ mice), mice were put on the same atherogenic regimen as previously described (Figure 1A). No differences were observed between WT and Klf4-KO mice in baseline weight, weight change, or total cholesterol levels after 24 weeks of atherogenic conditions (Supplemental Figure 5). Arteries from WT and Klf4-KO mice were harvested for scRNA-Seq and histological analyses. After filtering sequencing data for AdvSca1-SM cell–derived YFP^+^ cells following 16 weeks of atherogenic treatment, we observed major shifts in cell populations as a consequence of *Klf4* deletion in AdvSca1-SM cells (Figure 5A). In particular, we observed an increase in Fib_2 with a corresponding decrease in Fib_3 in arteries from the Klf4-KO mice compared with WT, precisely the opposite shift observed when comparing WT animals on a control diet with those on an atherogenic diet (Figure 5B). This increase in Fib_2 indicates that AdvSca1-SM cell–derived fibroblasts in Klf4-KO mice transition to a phenotype characterized by enriched ECM and collagen deposition. Additionally, comparing Fib_2 and Fib_3 populations between WT and Klf4-KO mice, cell populations from Klf4-KO mice expressed higher levels of collagen genes (*Col1a1* and *Col1a2*), further supporting a shift in Klf4-KO AdvSca1-SM cell–derived YFP^+^ fibroblasts toward a collagen deposition phenotype that potentially contributes to a protective fibrous cap or increased intraplaque collagen content (Figure 5C). Finally, there was a shift in the phenotype of AdvSca1-SM–derived transitional cells toward the mature SMC phenotype (Figure 5D), supporting a role in medial repair.

### Phenotypic modulation of AdvSca1-SM cells via Klf4 deletion modifies the fate of non–AdvSca1-SM–derived cells.

**Figure 6:**
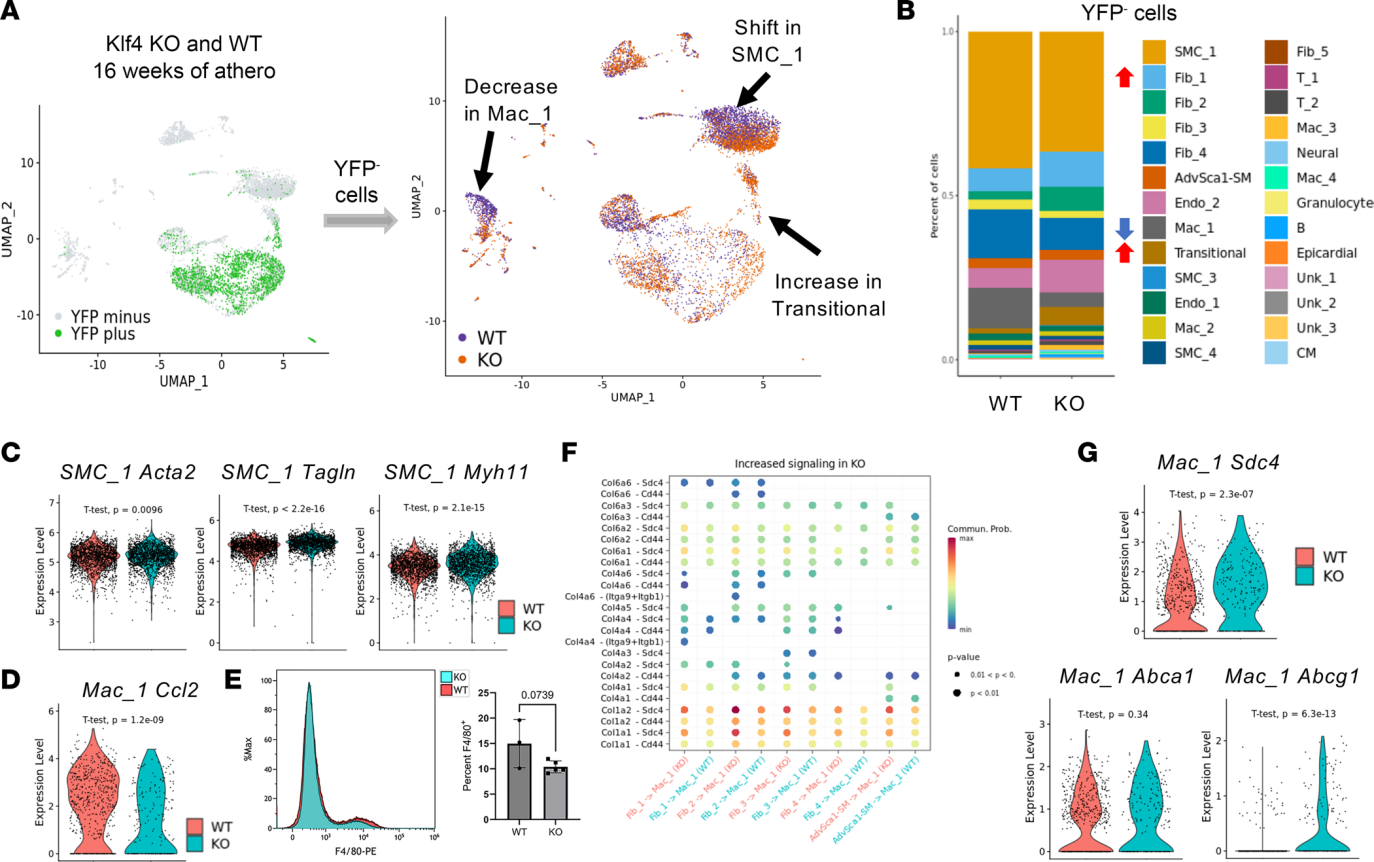
KLF4 depletion in AdvSca1-SM cells alters the fate of YFP^–^ non–AdvSca1-SM–derived cells. Arteries from 16-week WT and Klf4-KO AdvSca1-SM lineage atherogenic mice were processed for scRNA-Seq. (**A**) Left: UMAP projection of YFP^+^ and YFP^–^ cells from WT and Klf4 KO AdvSca1-SM cell lineage tracing mice. Right: UMAP projection showing the different fates of YFP^–^ cells in atherosclerosis as a consequence of depleting KLF4 in AdvSca1-SM cells. Arrows indicate major phenotypic shifts in KO mice, including changes to the SMC and macrophage clusters. (**B**) Stacked bar graph of YFP^–^ cells from WT and Klf4-KO mice after 16-week atherogenic diet. Arrows indicate increases (red) or decreases (blue) in the cell population as a result of KLF4 depletion. (**C**) Violin plot showing expression of SMC contractile genes (*Acta2*, *Tagln*, *Myh11*) in YFP^–^ cells in the major SMC cluster from Klf4-KO mice compared with WT mice. (**D**) Violin plot showing expression of *Ccl2* in YFP^–^ cells of the major macrophage cluster from Klf4-KO mice compared with WT mice. (**E**) Flow cytometry analysis of single-cell arterial digests from both WT and Klf4-KO mice after 24 weeks of atherogenic diet. (**F**) CellChat analysis was performed on the fibroblast/AdvSca1-SM and Mac_1 clusters. Bubble plot showing elevated levels of COL1A1/COL1A2 from the fibroblast clusters signaling to SDC4 in Mac_1 in the setting of atherosclerosis. (**G**) Violin plots show expression of *Sdc4* and *Abcg1* in Mac_1 from Klf4-KO mice in the setting of atherosclerosis. *Abca1* is not significantly different between the genotypes. Statistical analysis done with 2-tailed Student’s *t* test.

While we anticipated that KO of *Klf4* specifically in AdvSca1-SM cells would alter their differentiation patterns during atherosclerosis progression, we also observed substantial shifts in the phenotype of YFP^–^ non–AdvSca1-SM–derived cell populations in response to AdvSca1-SM cell Klf4 KO (Figure 6A). The most notable changes were in the gene expression profile of the major SMC cluster (SMC_1) as well as an overall decrease in the primary macrophage cluster (Mac_1) (Figure 6B). Further examination of the shift within the SMC_1 cluster revealed that SMC from Klf4-KO mice exhibited slightly elevated expression of smooth muscle contractile genes (Figure 6C). Investigation of Mac_1 revealed that macrophages from Klf4-KO mice expressed significantly lower levels of *Ccl2* (Monocyte Chemoattractant Protein-1 [MCP-1]), suggesting a reduced inflammatory phenotype and diminished recruitment of additional macrophages to the lesion (Figure 6D). Flow cytometry analysis of arteries from both WT and Klf4-KO mice after 24 weeks of atherogenic diet revealed a nonsignificant trend toward a decrease in F4/80^+^ macrophages in the whole vascular digest (Figure 6E). Since it has been well established that macrophages play an essential role in atherosclerosis progression, we sought to further define the phenotypic changes occurring in Mac_1 in the KO animals. Since *Klf4* was selectively deleted only in AdvSca1-SM cells and the Mac_1 population is almost entirely YFP (i.e., non–AdvSca1-SM cell–derived), we hypothesized a paracrine signaling axis resulted in the phenotypic shifts observed in Mac_1 macrophages from Klf4-KO mice. We investigated this possibility using CellChat, which infers cell-to-cell communications in scRNA-Seq data sets. We identified a high likelihood of fibroblast/AdvSca1-SM cell collagen-to-Mac_1 Syndecan 4 (SDC4) or CD44 signaling. Additionally, these signaling pathways were predicted to increase in KO animals (Figure 6F). Previous research has indicated that low levels of SDC4 in macrophages promotes atherosclerosis by polarizing macrophages toward a more proinflammatory state (25). This polarization was shown to occur through SDC4-mediated changes in ABCA1 and ABCG1 signaling, the ATP-binding cassette transporters essential for cholesterol efflux to HDL. Interestingly, we found that, compared with WT mice, macrophages from Klf4-KO mice expressed higher levels of *Sdc4* and *Abcg1* but not *Abca1* (Figure 6G). These findings highly support the concept that, not only are macrophages from WT animals more likely to recruit other macrophages via MCP-1, but they are also likely to be more proinflammatory and proatherogenic. Collectively, our data support a model in which manipulation of the phenotype of AdvSca1-SM cells results in autonomous effects on AdvSca1-SM cell differentiation and function as well as nonautonomous atheroprotective effects on additional cells of the atherosclerotic microenvironment.

### Klf4 KO in AdvSca1-SM cells does not reduce plaque burden, but it alters plaque complexity.

**Figure 7:**
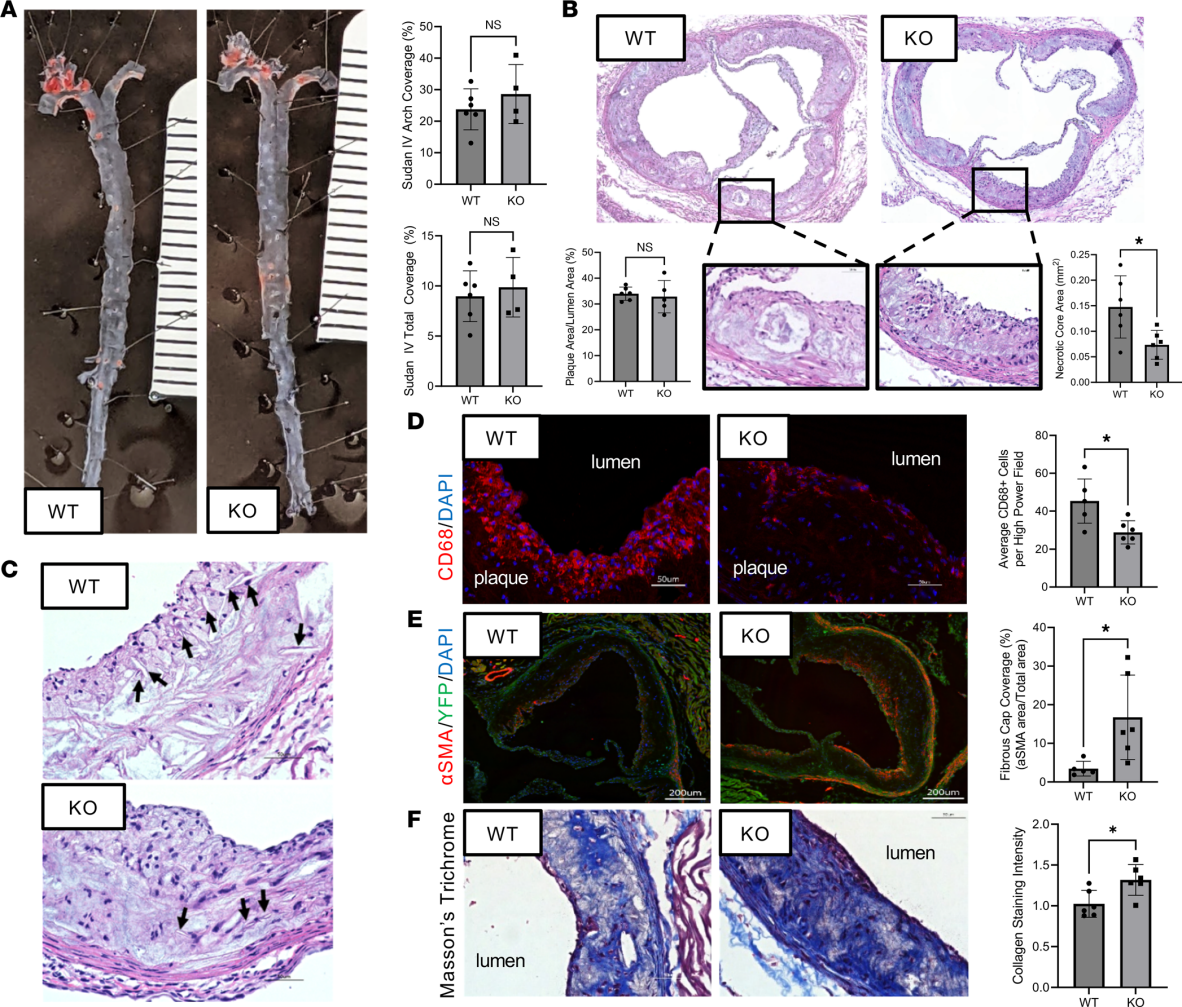
Depletion of KLF4 in AdvSca1-SM cells does not alter overall plaque burden but reduces late-stage plaque complexity. (**A**) The aortic arch and descending aorta from 24-week atherogenic WT (*n* = 6) and Klf4-KO (*n* = 4) mice were stained using Sudan IV to assess overall atherosclerotic plaque burden. Two-tailed Student’s *t* test analysis of staining is shown to the right. (**B**) Representative H&E images of aortic root cross sections from WT (*n* = 6) and Klf4-KO mice (*n* = 6). Insets show higher magnification of the plaque structure with significantly smaller necrotic cores in Klf4-KO mice compared with WT mice. Two-tailed Student’s *t* test analyses of plaque percent coverage and necrotic core area (*P* = 0.0221) are shown below. (**C**) Representative H&E images of stained aortic root cross sections from WT (*n* = 6) and Klf4-KO (*n* = 6) mice. Arrows indicate cholesterol clefts. (**D**) Representative immunofluorescence images of aortic root cross sections from WT (*n* = 5) and Klf4-KO (*n* = 6) mice for CD68 (red) and DAPI (blue). Two-tailed Student’s *t* test analysis of staining is shown to the right; *P* = 0.0117. (**E**) Representative immunofluorescence images of aortic root cross sections from WT (*n* = 5) and Klf4-KO (n = 6) mice for YFP (green), αSMA (red), and DAPI (blue). Two-tailed Student’s *t* test analysis of staining is shown to the right; *P* = 0.026. (**F**) Representative Masson’s trichrome images from WT (*n* = 6) and Klf4-KO (*n* = 6) mice aortic roots with collagen (blue), cytoplasm (pink), and cell nuclei (brown). Two-tailed Student’s *t* test analysis of staining is shown to the right; *P* = 0.0165. Scale bars: 50 μm, 200 μm (**E**).

To ascertain the physiological consequences of the AdvSca1-SM cell–specific Klf4 KO, we examined overall plaque burden. In the aortic root, aortic arch, and full vascular tree; there were no significant differences in total plaque burden between the WT and Klf4-KO mice at either early or late time points (Figure 7, A and B, and Supplemental Figure 7). However, there was a significant reduction in total necrotic core area in the plaques from Klf4-KO mice compared with WT mice, a change that would be indicative of a more stable plaque in humans (Figure 7B). Additionally, plaques from Klf4-KO mice showed fewer cholesterol clefts within the plaques (Figure 7C), supporting our earlier findings that suggest impaired cholesterol efflux to HDL in Mac_1 from WT compared with Klf4-KO mice. Our scRNA-Seq and flow data also revealed a nonsignificant trend toward an overall reduction in macrophages from Klf4-KO mice, but these approaches involved analysis of both diseased and nondiseased regions of the vessel (Figure 6B, scRNA-Seq and Figure 6E, flow). Since atherosclerosis is a focal disease, we also used an immunofluorescence approach to examine macrophage accumulation specifically in plaque regions. Compared with tissues from WT mice at baseline (Supplemental Figure 8), WT atherosclerotic tissues showed robust staining for CD68, a macrophage marker (Figure 7D), supporting macrophage accumulation in the setting of atherosclerosis. Importantly, macrophage infiltration was markedly reduced in atherosclerotic tissues from Klf4-KO mice (Figure 7D). While it was surprising that our scRNA-Seq data (Figure 2) do not show robust macrophage expansion in atherosclerotic vessels, this is most likely due to the focal nature of atherosclerosis, and as pointed out above, our approach used whole arteries with areas of uninvolved tissue, likely diluting the overall effect. Quantification of αSMA^+^ fibrous cap area in the aortic root revealed increased fibrous cap thickness in plaques from the Klf4-KO mice (Figure 7E). Finally, we investigated the plaque structure using Masson’s trichrome, which demonstrated increased collagen deposition in plaques from Klf4-KO mice compared with WT controls, supporting the shifts in fibroblast populations we identified in the scRNA-Seq experiments (Figure 7F). Collectively, these data show important alterations in the plaque composition of mice with AdvSca1-SM–specific Klf4 KO compared with WT controls.

## Discussion

**Figure 8:**
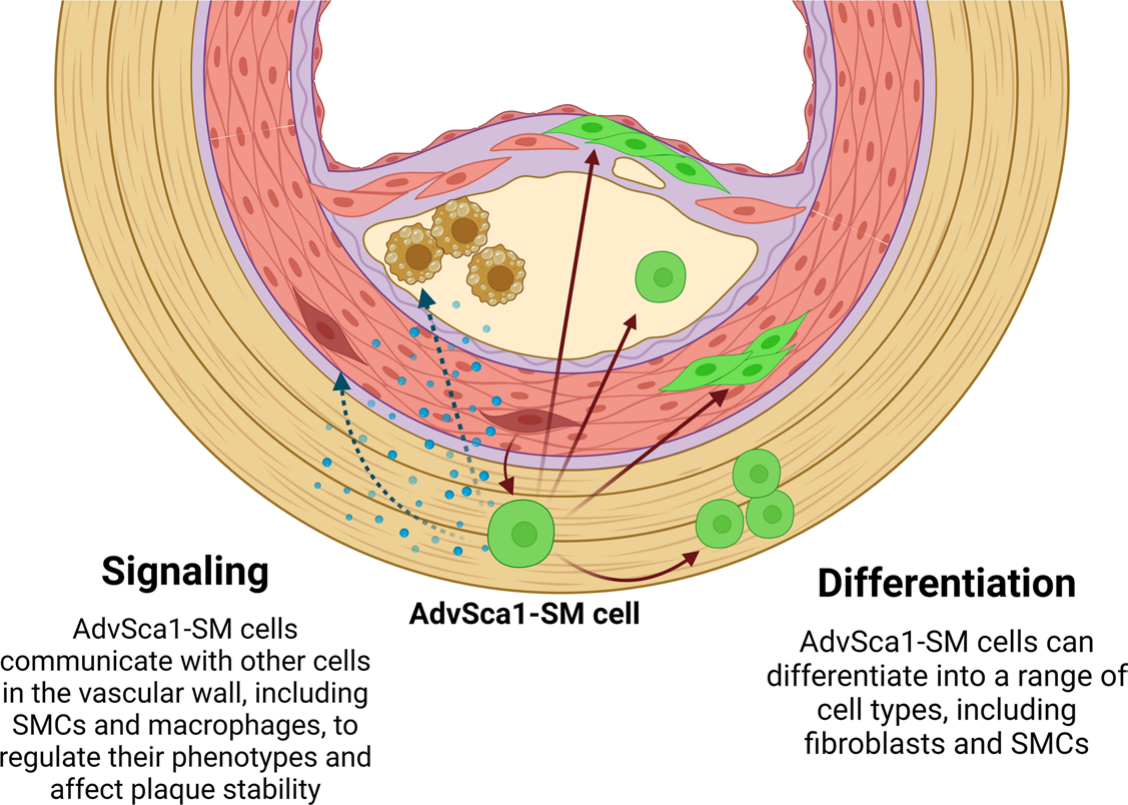
Schematic of the multifaceted roles of AdvSca1-SM cells in atherosclerosis. In the setting of atherosclerosis, AdvSca1-SM cells are capable of differentiating into a variety of cell types (primarily fibroblasts and SMC) and migrating throughout the vascular wall and the atherosclerotic plaque. In addition to directly differentiating into other cell types, AdvSca1-SM cells can signal to other cell types, including macrophages, fibroblasts, and SMC to alter their phenotypes. Genetic modulation of AdvSca1-SM cells drastically alters plaque composition complexity, indicating a central role for AdvSca1-SM cells in atherosclerosis.

In this study, we established the importance of an understudied cell population, AdvSca1-SM stem cells, to atherosclerosis progression. Consistent with our previous research in the setting of acute vascular injury, this is the first report to our knowledge demonstrating that AdvSca1-SM cells and AdvSca1-SM–derived cells are found throughout the vessel wall, contributing to pathological atherosclerotic lesions and adventitial remodeling. We also established the major differentiation pathways of AdvSca1-SM cells to fibroblasts and SMC, with minor contributions to other cell populations, including endothelial cells, macrophages, and adipocytes. AdvSca1-SM–specific genetic depletion of the reprogramming-associated transcription factor KLF4 drastically altered the differentiation trajectories of AdvSca1-SM cells as well as the phenotypes of other vascular cell types, including macrophages and SMC. Finally, we found that these autonomous and nonautonomous effects of KLF4 KO resulted in atheroprotection through decreased necrotic core size, increased fibrous cap thickness, increased intraplaque collagen, and decreased macrophage accumulation (Figure 8). It is important to note that there are key differences between human and murine atherosclerosis, particularly in that mouse plaques rarely rupture and therefore cannot be characterized as either stable or vulnerable (26). However, the changes in plaque composition as a result of AdvSca1-SM cell–specific KLF4 KO observed in this study would correspond to a more stable, protective plaque were it observed in humans.

Previous studies of the adventitia in the setting of atherosclerosis have focused on the role of the VV in regulating atherosclerosis via the “outside-in hypothesis” (6–9). Research has highlighted the importance of these microvessels by showing that neovascularization is correlated with plaque size while inhibition of plaque neovascularization reduced atherosclerosis progression (8, 27–30). In this study, we identified YFP^+^ AdvSca1-SM–derived cells surrounding adventitial microvessels, suggesting a role for AdvSca1-SM cells in VV expansion and potentially plaque neovascularization. However, it is important to note that the majority of adventitial AdvSca1-SM cells and AdvSca1-SM–derived cells were not found associated with the microvessels but were nonetheless greatly influenced plaque complexity through contribution to plaque core cells as well as fibrous cap cells. These findings support the importance of resident SMC-derived adventitial AdvSca1-SM stem cells in atherosclerosis.

Our finding that the primary differentiation trajectory for AdvSca1-SM cells in the setting of atherosclerosis is toward a fibroblast phenotype closely mirrors our previous findings in the setting of acute vascular injury (15, 16). Additionally, other groups using similar mouse models have indicated that these adventitial progenitor cells are involved in vascular calcification and neointima formation in the setting of chronic kidney disease and in the pathological profibrotic response in multiple organ systems (31, 32). However, in contrast to acute injury models in which perivascular fibrosis is detrimental, there is an important function for modulated SMC- and fibroblast-associated collagen deposition in protecting against necrotic core formation and plaque rupture (33). Importantly, our findings of increased collagen I and III expression by AdvSca1-SM–derived fibroblasts, as observed in Klf4-KO mice compared with WT mice, supports the concept that manipulating the phenotype of AdvSca1-SM cells confers a more stable/protective plaque phenotype, thereby making targeting of these cells an attractive therapeutic target.

A major focus of this study was the effect of *Klf4* deletion on AdvSca1-SM cell phenotype and function as well as on the plaque microenvironment. KLF4 has been widely recognized as one of the key transcription factors necessary for induction of pluripotent stem cells (induced pluripotent stem cells [iPSCs]) (34). In the vasculature, we demonstrated that KLF4 induction is essential for SMC reprogramming and maintenance of the AdvSca1-SM stem cell phenotype and that AdvSca1-SM–specific KLF4 depletion results in spontaneous differentiation toward a myofibroblast profibrotic phenotype (14, 15). Others have established that KLF4 is a potent repressor of the mature contractile phenotype and that KLF4 depletion in SMC results in a reduction in atherosclerotic plaque burden as well as aortic aneurysm formation (35, 36). Adding translational significance was the identification of *Klf4* by genome-wide association studies (GWAS) in human populations as a coronary artery disease risk locus (24, 37). However, whether KLF4 is protective or deleterious seems to be highly context dependent. Contrary to the previous findings in SMC, KLF4 depletion in endothelial and myeloid cells drives atherosclerosis progression (38, 39). In this study, we established that KLF4 in AdvSca1-SM cells is deleterious and that depletion results in more stable atherosclerotic plaques. This finding supports much of the prior research in vascular SMC but also serves to highlight the importance of elucidating the cell-specific effects of KLF4 in different disease processes.

One of the most unexpected findings was the ability of AdvSca1-SM cells to regulate the phenotype of plaque-associated macrophages. Our data suggest that cross communication may be a result of collagen produced by AdvSca1-SM cells and AdvSca1-SM–derived fibroblasts acting as a ligand for SDC4 on the surface of macrophages. A recent study from Hu et al. demonstrated that macrophage surface SDC4 is decreased in the setting of atherosclerosis and that this reduction in SDC4 is both proinflammatory and proatherogenic (25). It is believed that the proinflammatory macrophage phenotype is due to a decrease in ABCA1/ABCG1, which act as the rate-limiting steps for cholesterol efflux and reverse cholesterol transport (40, 41). Further highlighting the importance of the ABCA1/ABCG1 axis are studies showing that macrophage-specific deficiency of ABCA1 and ABCG1 promotes plaque inflammation and signals to bone marrow progenitors to produce more monocytes; it has also been demonstrated that ABCA1 and ABCG1 are major suppressors of plaque-associated leukocytosis, and this confers an atheroprotective effect (42, 43). These studies emphasize the central role of macrophage cholesterol efflux in the regulation of atherosclerosis. Importantly, our data with Klf4-KO mice compared with WT mice suggests that KO of *Klf4* in AdvSca1-SM cells results in enhanced collagen production and increased *Sdc4/Aba1/Abcg1* expression with a corresponding reduction in *Ccl2* expression, which likely contributes to the observed plaque protective effects.

One notable limitation in studying AdvSca1-SM cells is the dynamic nature of this cell population. AdvSca1-SM cells are poised to respond to changes in the vascular environment and therefore are capable of quickly differentiating away from their stemlike state. Similarly, SMC reprogramming to AdvSca1-SM cells is a dynamic process. Using our lineage tracing system, we were able to track these cells over time even as they underwent major cell transitions. However, due to the dynamic SMC–to–AdvSca1-SM cell transition, as shown in both our previous work and this study, newly generated AdvSca1-SM cells will be formed but will not be labeled with the AdvSca1-SM reporter, nor will KLF4 be depleted in the case of Klf4-KO mice (14). This cell turnover is likely to have diluted some of the findings in this study, and future investigation into the timing and frequency of these reprogramming events is likely warranted. Despite these technical concerns, the dynamic nature of the ongoing reciprocal SMC–to–AdvSca1-SM transitions identified by RNA velocity analysis suggest that understanding the mechanisms involved in these transitions might provide sensitive therapeutic targets.

To summarize, in this study, we used a combination of histological and scRNA-Seq approaches to define the multifaceted contributions of AdvSca1-SM cells to atherosclerosis, both through direct differentiation into other cell types and through signaling to SMC and macrophages. Additionally, we revealed the potential for modulation of these resident vascular progenitor cells to regulate atherosclerotic plaque complexity. Collectively, this work emphasizes the continuing importance of research into the vascular adventitia as a driver of atherosclerosis and a potential therapeutic target.

## Methods

### Experimental model and subject details

#### Mice.

This research utilized a variety of transgenic mice generated from widely available strains. Gli1-Cre^ERT^ (JAX 007913), Myh11-Cre^ERT^ (Stephan Offermanns, Max-Planck-Institute for Heart and Lung Research, Germany; now available at JAX 019079), and Rosa26-YFP (JAX 006148) mice were all obtained from The Jackson Laboratory. Klf4-floxed mice were obtained from Klaus H. Kaestner (University of Pennsylvania, Philadelphia, Pennsylvania, USA). All mice currently reside as colonies in-house.

Lines generated by breeding included (a) Gli1-Cre^ERT^/Rosa26-YFP (AdvSca1-SM lineage tracing mouse [WT]), (b) Gli1-Cre^ERT^/Rosa26-YFP/Klf4^fl/fl^ (AdvSca1-SM lineage tracing mouse with AdvSca1-SM specific deletion of Klf4 [Klf4-KO]); and (c) Myh11-Cre^ERT^/Rosa26-YFP (SMC lineage tracing mouse).

All mice bred to Gli1-Cre^ERT^ transgenic mice are inducible systems for AdvSca1-SM reporter knock-in and/or AdvSca1-SM–specific Klf4 KO; mice bred to Myh11-Cre^ERT^ are inducible systems for SMC reporter knock-in. Experimental mice were maintained as heterozygous for the Gli1-Cre^ERT^ or hemizygous for Myh11-Cre^ERT^ and homozygous for YFP reporter and Klf4 flox. Age-matched male and female mice were used for all AdvSca1-SM lineage mouse experiments, with tamoxifen injections beginning between 6 and 8 weeks of age and initiation of atherogenic or control treatment regimens beginning between 8 and 10 weeks of age. Only male mice were used for the SMC lineage tracing mouse experiments, as the Cre was inserted on the Y chromosome. Weight was measured at the beginning and end of the studies to identify any outliers. Starting weight was not significantly different between WT and KO mice at baseline, nor was there a difference between the genotypes in weight change during the experiments (Supplemental Figure 5).

Experimental mice were housed in the AAALAC (American Association for Accreditation of Laboratory Animal Care)-accredited RC-2 vivarium at the University of Colorado Anschutz Medical Campus. The vivarium was maintained at standard subthermoneutral temperatures (22°C–26°C) with a 12-hour light-dark cycle. All animals had ad libitum access to both food and water. Atherogenic mice received Envigo TD.02028 (42.6% fat, 1.3% cholesterol, and 0.5% cholic acid) or Research Diets D05060402 (42.8% fat, 1.5% cholesterol, and 0.5% cholic acid), whereas control animals received the standard diet Envigo 2920x (16% fat); all diets were irradiated prior to transfer into the animal facility. Animal health was monitored daily by veterinary staff, and animals were humanely euthanized per protocol in the event of any significant illness or injury. Cages were changed every 2 weeks and contained enrichment materials, including nesting material (e.g., Nestlet, brown shredded paper, paper towels) and/or cage furniture. Animals on atherogenic diet received front and rear nail trims as needed to minimize scratch trauma resulting from diet-induced dermatitis.

### Mouse genotyping

All mice used in this project were bred and maintained by our lab in the University of Colorado RC-2 Vivarium. At the time of weaning, mice were ear tagged and ear snipped for genotyping and future identification. Ear snips were then processed using Extracta DNA Prep for PCR-Tissue (QuantaBio, 95091-025) per manufacturer protocol. DNA samples were stored at –20°C until ready for genotyping.

Prior to use in experiments, mice were genotyped using the recommended primers and PCR programs from The Jackson Laboratory. For each primer set, a master mix was prepared with 10 μL AccuStart II PCR SuperMix (QuantaBio 95137), 1 μL of each primer (listed below, diluted 1:10 in molecular grade water for use), and molecular grade water to bring reaction total up to 17 μL per sample. Master mix was loaded into PCR strip tubes, and 3 μL of the appropriate mouse sample, positive control, or negative control were added. Strip tubes were spun down and then run on the appropriate PCR program as recommended by The Jackson Laboratory on a Bio-Rad T100 Thermocycler. PCR products were run on a 2% agarose gel with 5 μL ethidium bromide per 100 mL of agarose/TAE mix. Gels were imaged using an AlphaImager Mini. Each mouse was genotyped with the Gli1-Cre, Cre, Rosa26-YFP, Myh11-Cre, and Klf4 flox (as appropriate) primer sets.

### In vivo mouse procedures

Prior to experimentation, all mice (both on atherogenic and standard diets) were injected with tamoxifen to activate Cre recombinase. Stock tamoxifen solution was prepared using 400 mg tamoxifen (MilliporeSigma, T5648-5G), 2 mL 100% ethanol, and 38 mL corn oil. The solution was incubated in a 60°C bead bath with intermittent vortexing for 3–5 hours, until the tamoxifen was fully dissolved. Tamoxifen solution was then passed through a 0.2 mm syringe filter and stored at –20°C. To activate Cre recombinase, AdvSca1-SM lineage mice between the ages of 6 and 8 weeks received 12 consecutive days of i.p. injections, a dosing regimen validated by our lab’s previous research; SMC lineage mice received 7 consecutive days of injections. Each injection was 150 μL, delivered through a 25G syringe (1.5 mg/day), and the side of injection was alternated daily to minimize irritation and trauma. Mice were weight stable throughout the injections (Supplemental Figure 1).

Following tamoxifen injections, mice were weighed to obtain baseline metrics. Mice randomized to the nonatherogenic arm of the study were then placed in a new group housing cage and maintained on standard chow Envigo 2920x (16% fat). Mice randomized to atherogenic treatment received a retroorbital injection of AAV bearing a mutant gain-of-function PCSK9 (Vector Biolabs, AAV8-D377Y-mPCSK9, Addgene 58376). PCSK9 is involved in clearance of LDL-cholesterol (LDL-C) from the bloodstream by mediating LDL receptor (LDLR) internalization and lysosomal degradation. When coupled with a high-fat diet, injection of 1 × 10^11^ gene copies (gc) per mouse of AAV-m-PCSK9 has been shown to induce hypercholesterolemia and atherosclerotic lesion formation in mice without ApoE^–/–^ or LDLR^–/–^ mutations (17). The AAV stock was diluted in sterile saline such that each mouse received 1 × 10^11^ gc delivered in 200 μL of solution through a 28G syringe. The retroorbital injections were conducted under anesthesia per institutional protocol (induction at 3%–5% isoflurane and maintenance on the nose cone at 1.5%–3% isoflurane). In a subset of mice, retroorbital blood draws were used to collect plasma at the time of PCSK9 injection and throughout the experiment. Following injection of the virus and/or retroorbital blood draws, animals were treated with Proparacaine HCl 0.5% ophthalmic eye drops (Bausch & Lomb, 24208073006). Animals were observed for full recovery from the procedure and were then placed in a new group housing cage and given an atherogenic diet. A second dose of the AAV-m-PCSK9 was delivered after 2 weeks to ensure adequate viral load to induce hypercholesterolemia and lesion formation, and an additional dose was given at 16 and 24 weeks for mice randomized to longer endpoints. Mice were maintained under these conditions until their assigned endpoint was reached (8, 16, 24, or 28 weeks); they were then were humanely euthanized.

### Lectin staining

A subset of mice was injected with fluorescently labeled lectin in vivo to identify functional vasculature. The B4 isolectin has high affinity for terminal α-D-galactosyl residues and binds to endothelial cells. The lectin solution (100 μL *Griffonia simplicifolia* lectin I [GSL I-B4], DyLight 594 + 100 μL sterile saline) was retroorbitally injected into mice (200 μL solution per mouse) and allowed to circulate for 5 minutes prior to sacrifice to label the vasculature.

### Mouse euthanasia and tissue harvest

Mice were euthanized and harvested according to institutional protocol either at their assigned endpoint or in the case of significant injury/illness. Specifically, mice were exposed to an overdose of isofluorane using the drop technique and left in the chamber until cessation of respiration. At that point, mice were removed from the chamber, and cervical dislocation was performed as a secondary method of euthanasia (bilateral thoracotomy and exsanguination were also performed later during the harvest). Using a dissecting microscope, the abdominal cavity was opened, the diaphragm was cut, and the ribcage was removed. Blood was collected via cardiac puncture using a 25G syringe and was then stored on ice for up to 1 hour during the harvest. Following exsanguination, mice were perfused with 10 mL of PBS/heparin (MilliporeSigma, H3393; diluted to 0.08 KU/mL in PBS).

For tissues being collected for histology, mice were then perfused with 10 mL of 4% PFA in PBS. The heart, combined aortic arch/brachiocephalic artery (BCA)/carotid arteries, and the descending aorta were then microdissected out of the animal and placed in Eppendorf tubes containing 4% PFA. These samples were placed at 4°C overnight to ensure adequate fixation. For tissues being collected for flow cytometry or scRNA-Seq, the heart, aortic arch/BCA/carotids, and descending aorta (for flow cytometry only) were then microdissected out of the animal and placed in dishes containing 10% FBS MEM to maintain cell viability. These dishes were left on ice for the duration of the harvest.

### Mouse serum analysis

For serum collection, tubes of blood were removed from the ice and left at room temperature for 30 minutes to clot; then, the clots were dislodged using p200 pipette tips. For plasma collection, retroorbital blood draws were performed using microhematocrit capillary tubes (Thermo Fisher Scientific, 22-362566) and collected in heparinized microtainers (BD Biosciences, 365965). Blood samples were spun down in a tabletop centrifuge for 10 minutes at 4°C and 10,000*g*. The supernatant (serum or plasma) was removed from the tube and transferred to a new Eppendorf for storage at –80°C until analysis.

Serum was analyzed in batches for total cholesterol to confirm that animals in the atherogenic arm of the study responded to the AAV-m-PCSK9 and high-fat/high-cholesterol diet. FujiFilm Wako Diagnostics kits for Cholesterol E (no. 999-02601) were used according to manufacturer recommendations. Samples were diluted down 1:10 in sterile water so they fell on the standard curve. All tests were completed in triplicate.

### Histology and immunofluorescence

Tissues collected for histology were stored at 4% PFA at 4°C overnight to allow for tissue fixation. After 24 hours, tissues were removed from the PFA, rinsed with PBS, and then transferred to 30% sucrose/PBS. The sucrose/PBS incubation was for a minimum of 24 hours at 4°C to ensure adequate cryoprotection. Tissues were then embedded in Tissue-Tek OCT Compound (Sakura) and frozen per manufacturer recommendations. Tissue blocks were stored at –80°C until sectioning. Serial sections 6 μm thick were collected from each block for use in histology and immunofluorescent/RNAscope microscopy studies.

Slides were stained with H&E and/or Masson’s trichrome by the Anschutz Pathology Shared Resource Research Histology group. For fluorescence imaging, sections were rehydrated, permeabilized, blocked with 3% horse serum in PBS, and double or triple stained for combinations of YFP, SCA1, SMC markers (αSMA), macrophage markers (CD68), adipocyte markers (FABP44), erythrocyte markers (TER-119), and others. IgG control staining is shown in Supplemental Figure 9. Slides were then imaged using a Keyence BZ-X710 microscope and BZ-X Viewer image acquisition software. For imaging, exposure and gain were consistent throughout an experiment, and signal/noise ratios were maximized. Quantification of plaque characteristics was performed in a blinded fashion by a minimum of 2 independent investigators using ImageJ/Fiji software (NIH). Plaque area/necrotic core area was measured from H&E-stained sections, plaque collagen density was measured from Masson’s trichrome–stained sections using the Colour Deconvolution tool, and CD68^+^ cells/αSMA^+^ fibrous cap area were measured from IF stained sections.

### RNAscope

RNAscope was used to detect colocalization of fibroblast markers (Lum) with YFP^+^ cells in the aortic root sections. Positive and negative control probes were employed. For detection of Lum, slides were prepared according to the RNAscope RED Assay and Immunofluorescence technical note (322350-TN/Rev A/Draft Date 06052017) and RNAscope 2.5 HD Detection Reagent RED User Manual (322360-USM/Rev/Draft Date 11052015). Following RNAscope signal detection, slides were washed with PBS and stained with a FITC-conjugated anti-GFP antibody following standard immunofluorescence staining. Negative control probe is shown in Supplemental Figure 9.

### Sudan IV staining

Sudan IV staining was used to assess lipid burden in whole aortas. The Sudan IV staining solution (Sigma Aldrich 198102-25g) included 0.5 gm Sudan IV powder, 25 mL acetone, 17.5 mL 100% ethanol, and 7.5 mL DI water. Ingredients were mixed, left to rest for ~1 hour, and then filtered through a 0.2 μm Nalgene Rapid-Flow unit. The vascular tree (aortic arch through the common iliac artery) was prepared for staining by trimming fat from the perivascular area before being cut open longitudinally to expose the luminal surface. Vessels were rinsed in 70% ethanol for 30 seconds and were then placed in a 12-well plate and covered with Sudan IV solution. Samples were incubated at room temperature on a rocker for 30 minutes before being rinsed twice with 80% ethanol to destain the nonplaque regions. Any remaining perivascular fat was removed; then, vessels were pinned open for imaging of plaque burden and quantification using ImageJ/Fiji.

### Single-cell digests

Tissues collected for flow cytometry were maintained in dishes containing 10% FBS MEM on ice for the duration of the mouse harvest to maintain cell viability. Following harvest, tissues were rinsed in HBSS to remove any serum from the samples, since serum can interfere with the tissue digest. Digest solution was made from 5 mg/mL Elastase (Worthington), 0.2 mg/mL Soybean Trypsin inhibitor (MilliporeSigma), and 3.2 mg/mL collagenase II dissolved in HBSS and filtered through a 0.2 mm syringe filter. Individual tissues were minced using dissection scissors in Eppendorf tubes with 500 μL digestion buffer before being incubated in a 37°C incubator for about 1 hour to create a single-cell suspension. Samples were removed from the incubator at approximately 10-minute intervals and pipetted with a p1000 tip to facilitate tissue digestion.

At the end of the digest period, samples were spun down for 12 minutes at 172*g* (4°C) to pellet the single cells. Supernatant was removed, and cells were resuspended in FA3 Buffer. The FA3 buffer was prepared with 1× PBS, 1 mM EDTA, 25 mM HEPES (pH 7.0), and 1% FBS and was then sterile filtered. Samples were centrifuged again with the same parameters, resuspended FA3, then filtered through 70 mm FLOWMI cell strainers.

### Flow cytometry

Following filtration, samples were spun down again, and pellets were resuspended in low volume FA3. In total, 100 μL of each sample was placed in a 96–deep well U-bottom plate, with excess samples pooled for use as staining controls (single color, FMO, isotype, and full stain). Plates were spun down (at 4°C for 12 minutes at 600*g* for every time unless noted), and 10 μL of FC block (diluted 1:10 in FA3) was added to each sample well and incubated at room temperature. After a 10-minute incubation, 100 μL of antibody cocktails for cell-surface markers were added to the samples before being incubated at room temperature for 30 minutes, shielded from light. After 30 minutes, plates were spun down at 600*g* (other parameters remained the same), supernatant was removed, and 150 μL Fixation Buffer (Invitrogen, 88-8824-00) was added to each well. Plates were incubated at 4°C for 1 hour away from light. Plates were spun down again at 600*g*, supernatant was removed, and samples were resuspended in 600 μL FA3 buffer for overnight storage. Plates were wrapped with Parafilm and aluminum foil to protect against evaporation and light exposure; they were then were stored at 4°C overnight.

The following day, plates were spun down at 600*g*, supernatant was removed, and samples were rinsed with sterile PBS. After an additional spin at 600*g* and decanting of supernatant, 100 μL of antibody cocktails for intracellular markers were added to each well. Plates were incubated at 4°C for 2 hours away from light. Plates were then spun again at 600*g*, supernatant was removed, and samples were incubated with Permeabilization Buffer (Invitrogen, 88-8824-00) for 30 minutes. This process was repeated to allow for a second rinse. After the final spin, cells were resuspended in FA3 and transferred to 1.2 mL microdilution tubes. These samples were then kept at 4°C and shielded from light until ready to process on the Gallios or Gallios 561.

### scRNA-Seq

For scRNA-Seq, single-cell digests were prepared from the aortic root, aortic arch, BCA, and carotid arteries as described above. To have sufficient cells for the capture/sequence, 3 mice per condition were pooled into a single sample. Following preparation of single-cell digests, each sample was stained with DAPI and flow sorted to remove RBCs, cell debris, and dead cells. Cells were also sorted into YFP^+^ and YFP^–^ samples for each condition (Supplemental Figure 3). Samples were spun down and resuspended in 2% FBS PBS to a target concentration of 1,000 cells/μL. Samples were submitted to the Anschutz Genomics Shared Resource, and a total of 5,000 cells per sample was captured and sequenced at a depth of 5,000 reads per cell using the 10X Genomics platform. Sequencing data were processed through the Cell Ranger pipeline with custom build reference genome (Ensembl GRCm39 release 104) containing eYFP ORF sequence. scRNA-Seq data were analyzed using Seurat and other R packages; code for this analysis has been deposited in the Weiser-Evans Lab GitHub (https://github.com/weiserevanslab/adubner). The Seurat object was analyzed with scDblFinder to detect likely cell doublets, and these were removed from the data set (Supplemental Figure 4A). nFeature (number of unique genes per cell), nCount (total number of molecules per cell), and percent.mito (percent of reads mapped to mitochondrial genome) were plotted and used to determine filtering criteria (nFeature_RNA > 500, nFeature_RNA < 6,500, nCount_RNA > 500, nCount_RNA < 45,000, percent.mito < 15), as shown in Supplemental Figure 4, B–D. A DimPlot of all samples showing distribution of cells from each origin sample as well as plots of time point and atherosclerosis versus control treatment conditions and a confusion matrix were used to confirm that batch effects were not observed and that no batch correction or integration was needed for this data set (Supplemental Figure 4, E and F). Seurat object was also passed through the Harmony batch integration algorithm (https://portals.broadinstitute.org/harmony/) and processed in parallel to the primary Seurat object to ensure our findings were not a result of batch effects (data not shown). Additional packages utilized in this analysis were dittoSeq to generate stacked bar plots, enrichR for GO and KEGG enrichment analysis, Ggpubr for statistical analysis of violin plots, and CellChat for inference and analysis of cell-to-cell communication. RNA velocity analysis was performed using scvelo in a Python environment. Top markers for each cluster can be found in Supplemental Table 1 (top genes per cluster in all conditions, all genotypes), Supplemental Table 2 (top genes per cluster in WT mice under all conditions), and Supplemental Table 3 (top genes per cluster in KO mice under all conditions).

### Materials availability

The materials generated from this work may be shared with the scientific community after appropriate invention disclosure(s) and adequate protection has been achieved by the University of Colorado’s Technology Transfer Office. Samples of research materials will be made available to the research community following completion of the University of Colorado’s Material Transfer Agreement.

### Statistics

All experimental data were evaluated for normality through comparison of median and means, SDs, and through formal normality tests (Shapiro-Wilk). The appropriate statistical analysis was used, dependent on the outcome of the normality analyses. Normally distributed continuous data were analyzed using 2-tailed Student’s *t* tests, whereas skewed data were analyzed using the nonparametric Mann-Whitney *U* test. The α level was set to 0.05, and statistical tests were completed using GraphPad Prism 9; statistical analysis of violin plots was completed using the Ggpubr R package. Bar graph data were plotted as mean ± SD, with all individual values displayed.

### Study approval

All mice for this project were approved by CU Anschutz IACUC, protocol no. 00066. Animal numbers were approved by the University of Colorado Anschutz Medical Campus Animal Institute Committee.

### Data and code availability

This research generated data from experiments using genetic mouse models. Data sets for scRNA-Seq have been deposited in the NCBI Gene Expression Omnibus site (https://www.ncbi.nlm.nih.gov/geo/; Accession no. GSE244439). scRNA-Seq data were analyzed with using Seurat and other R packages; code for this analysis has been deposited in the Weiser-Evans Lab GitHub (https://github.com/weiserevanslab/adubner; Commit ID 5454b4e). Supporting data values for all graphs are available in the Supporting Data Values supplemental material. Additional raw data presented in this manuscript can be obtained upon reasonable request to the lead contact.

## Author contributions

AMD and MCMWE designed the studies. AMD, SL, AJJ, KAS, and MFM performed the experiments. TH and TN assisted with mouse colonies and microscopy imaging. AMD and SL performed the bioinformatics analysis. KSM provided reagents and training for plaque analysis. AMD and MCMWE wrote the manuscript. SL, AJJ, KAS, MFM, TH, TN, RAN, KSM, and MWM edited the manuscript.

## Supplementary Material

Supplemental data

Supporting data values

## Version 1 11/22/2023

Electronic publication

## References

[1] Libby P (2019). The changing landscape of atherosclerosis. Nature.

[2] https://www.who.int/news-room/fact-sheets/detail/cardiovascular-diseases-(cvds).

[3] Gimbrone MA, García-Cardeña G (2016). Endothelial cell dysfunction and the pathobiology of atherosclerosis. Circ Res.

[4] Borén J, Williams KJ (2016). The central role of arterial retention of cholesterol-rich apolipoprotein-B-containing lipoproteins in the pathogenesis of atherosclerosis: a triumph of simplicity. Curr Opin Lipidol.

[5] Tabas I, Bornfeldt KE (2016). Macrophage phenotype and function in different stages of atherosclerosis. Circ Res.

[6] Mulligan-Kehoe MJ, Simons M (2014). Vasa vasorum in normal and diseased arteries. Circulation.

[7] Maiellaro K, Taylor WR (2007). The role of the adventitia in vascular inflammation. Cardiovasc Res.

[8] Moulton KS (2003). Inhibition of plaque neovascularization reduces macrophage accumulation and progression of advanced atherosclerosis. Proc Natl Acad Sci U S A.

[9] Kawabe J ichi, Hasebe N (2014). Role of the vasa vasorum and vascular resident stem cells in atherosclerosis. Biomed Res Int.

[10] Gräbner R (2009). Lymphotoxin beta receptor signaling promotes tertiary lymphoid organogenesis in the aorta adventitia of aged ApoE-/- mice. J Exp Med.

[11] Majesky MW (2011). The adventitia: a dynamic interface containing resident progenitor cells. Arterioscler Thromb Vasc Biol.

[12] Majesky MW (2015). Adventitia and perivascular cells. Arterioscler Thromb Vasc Biol.

[13] Jolly AJ (2022). Heterogeneous subpopulations of adventitial progenitor cells regulate vascular homeostasis and pathological vascular remodelling. Cardiovasc Res.

[14] Majesky MW (2017). Differentiated smooth muscle cells generate a subpopulation of resident vascular progenitor cells in the adventitia regulated by Klf4. Circ Res.

[15] Lu S (2020). Smooth muscle–derived progenitor cell myofibroblast differentiation through KLF4 downregulation promotes arterial remodeling and fibrosis. JCI Insight.

[16] Jolly AJ (2023). Redistribution of the chromatin remodeler Brg1 directs Smooth muscle–derived adventitial progenitor-to-myofibroblast differentiation and vascular fibrosis. JCI Insight.

[17] Goettsch C (2016). A single injection of gain-of-function mutant PCSK9 adeno-associated virus vector induces cardiovascular calcification in mice with no genetic modification. Atherosclerosis.

[18] Bjørklund MM (2014). Induction of atherosclerosis in mice and hamsters without germline genetic engineering. Circ Res.

[19] Bergen V (2020). Generalizing RNA velocity to transient cell states through dynamical modeling. Nat Biotechnol.

[20] Li Q (2022). Single-cell RNA sequencing in atherosclerosis: Mechanism and precision medicine. Front Pharmacol.

[21] Yu L (2023). Heterogeneity of macrophages in atherosclerosis revealed by single-cell RNA sequencing. FASEB J.

[22] Wirka RC (2019). Atheroprotective roles of smooth muscle cell phenotypic modulation and the TCF21 disease gene as revealed by single-cell analysis. Nat Med.

[23] Pan H (2020). Single-cell genomics reveals a novel cell state during smooth muscle cell phenotypic switching and potential therapeutic targets for atherosclerosis in mouse and human. Circulation.

[24] Alencar GF (2020). Stem cell pluripotency genes Klf4 and Oct4 regulate complex SMC phenotypic changes critical in late-stage atherosclerotic lesion pathogenesis. Circulation.

[25] Hu J (2022). A reduction of syndecan-4 in macrophages promotes atherosclerosis by aggravating the proinflammatory capacity of macrophages. J Transl Med.

[26] Daugherty A (2017). Recommendation on design, execution, and reporting of animal atherosclerosis studies: a scientific statement from the American Heart Association. Arterioscler Thromb Vasc Biol.

[27] Fleiner M (2004). Arterial neovascularization and inflammation in vulnerable patients: early and late signs of symptomatic atherosclerosis. Circulation.

[28] Kumamoto M (1995). Intimal neovascularization in human coronary atherosclerosis: its origin and pathophysiological significance. Hum Pathol.

[29] Moulton KS (1999). Angiogenesis inhibitors endostatin or TNP-470 reduce intimal neovascularization and plaque growth in apolipoprotein E-deficient mice. Circulation.

[30] Langheinrich AC (2006). Correlation of vasa vasorum neovascularization and plaque progression in aortas of apolipoprotein E(-/-)/low-density lipoprotein(-/-) double knockout mice. Arterioscler Thromb Vasc Biol.

[31] Kramann R (2016). Adventitial MSC-like cells are progenitors of vascular smooth muscle cells and drive vascular calcification in chronic kidney disease. Cell Stem Cell.

[32] Kramann R (2015). Perivascular Gli1+ progenitors are key contributors to injury-induced organ fibrosis. Cell Stem Cell.

[33] Bentzon JF (2014). Mechanisms of plaque formation and rupture. Circ Res.

[34] Takahashi K, Yamanaka S (2006). Induction of pluripotent stem cells from mouse embryonic and adult fibroblast cultures by defined factors. Cell.

[35] Shankman LS (2015). KLF4-dependent phenotypic modulation of smooth muscle cells has a key role in atherosclerotic plaque pathogenesis. Nat Med.

[36] Salmon M (2013). KLF4 regulates abdominal aortic aneurysm morphology and deletion attenuates aneurysm formation. Circulation.

[37] Van Der Harst P, Verweij N (2018). Identification of 64 novel genetic loci provides an expanded view on the genetic architecture of coronary artery disease. Circ Res.

[38] Zhou G (2012). Endothelial Kruppel-like factor 4 protects against atherothrombosis in mice. J Clin Invest.

[39] Sharma N (2012). Myeloid Krüppel-like factor 4 deficiency augments atherogenesis in ApoE-/- mice--brief report. Arterioscler Thromb Vasc Biol.

[40] Cuchel M, Rader DJ (2006). Macrophage reverse cholesterol transport: key to the regression of atherosclerosis?. Circulation.

[41] Poznyak AV (2022). Cholesterol transport dysfunction and its involvement in atherogenesis. Int J Mol Sci.

[42] Westerterp M (2013). Deficiency of ATP-binding cassette transporters A1 and G1 in macrophages increases inflammation and accelerates atherosclerosis in mice. Circ Res.

[43] Yvan-Charvet L (2010). ATP-binding cassette transporters and HDL suppress hematopoietic stem cell proliferation. Science.

